# Toward brain-trained humanoid robots

**DOI:** 10.3389/fnbot.2026.1903423

**Published:** 2026-09-28

**Authors:** Maël Donoso

**Affiliations:** Ouroboros Neurotechnologies, Lausanne, Switzerland

**Keywords:** brain-trained AI, brain-trained humanoid robots, embodied alignment, embodied reasoning, embodiment-dependent neural signals, humanoid robots, human-robot interaction, neuroimaging

## Abstract

A growing corpus of experimental research demonstrates that neuroimaging data can be leveraged to improve the performance of artificial intelligence models, including reinforcement learning models in robotics. However, most existing studies have focused on vision, audition, language, or reward and error signals recorded while the human brain acts as an observer. In this paper, we argue that humanoid robots, although not equivalent to humans, could offer a unique opportunity to use the neural signals associated with touch, or other embodiment-dependent neural signals. As a result, humanoid robots could significantly expand the range of brain regions and functional networks that could be targeted for brain-trained artificial intelligence, and open new horizons in terms of alignment and reasoning. We discuss the improvements that neuroimaging data could potentially unlock in the context of sensorimotor tasks, spatial navigation, sensorimotor integration, embodied alignment, embodied reasoning, and social integration, as well as the broader challenges and opportunities related to brain-trained humanoid robots.

## 1 Introduction

Do androids dream of electric brains? While the human brain is usually viewed as an inspiration for artificial intelligence (AI) systems, a growing corpus of experimental research demonstrates that neuroimaging data can also be directly leveraged to improve the performance of classical machine learning, deep learning, or reinforcement learning models for a variety of tasks. In the visual domain, studies have used functional magnetic resonance imaging (fMRI) data (Fong et al., 2018; Lu and Wang, 2025) and electroencephalography (EEG) data (Spampinato et al., 2017) to optimize models for vision-related tasks, all the while providing new insights into the cognitive processes of the human brain (Hansen et al., 2025). In the auditory domain, studies have similarly exploited fMRI data to optimize models for audition-related tasks (Freteault et al., 2025), as well as to improve their performance on downstream semantic tasks (Moussa et al., 2025). In the linguistic domain, fMRI and magnetoencephalography (MEG) data have been used to align language models with human semantic representations (Schwartz et al., 2019), including in multilingual contexts (Negi et al., 2025). Nevertheless, one of the most promising applications for brain-trained AI could be robotics, where foundational research has demonstrated that reward and error signals obtained through EEG can serve to guide reinforcement learning models (Iturrate et al., 2010). Subsequent studies have further confirmed that EEG data can improve the performance of robots (Kim et al., 2017), accelerate their training in complex virtual navigation games (Xu et al., 2021), and help them achieve high success rates in simple physical games (Kar et al., 2022).

The recently proposed framework of brain-trained foundation models suggests that a diversity of additional neural signals could potentially be leveraged to improve the performance of AI systems (Donoso, 2026b). According to this framework, if *B*(*t*) represents the brain activity of a human subject over time, *A*(*t*) the observable actions of the subject, and B^*^(*t*) the available neuroimaging data for the subject, then it is hypothesized that: *A*(*t*) could be considered a subset or projection of *B*(*t*), since all voluntary actions are expected to emerge from the brain activity; B^*^(*t*) could include elements of *B*(*t*) that are not included in *A*(*t*). This second assumption suggests that neuroimaging data could serve as a valuable training resource for AI systems, since it could provide insights into human cognition that would not be accessible from behavioral data alone. However, until now, empirical exploration of the potential of human brain data for AI systems has been heterogeneous, with most research efforts focusing on vision, audition, language, or observational reward and error signals—cognitive processes that are arguably less dependent on embodiment than, for example, tactile perception or motor action. Remarkably, despite the extensive set of brain regions and functional networks engaged in more embodiment-dependent cognitive processes, such as touch, this particular category of neural signals appears to be essentially missing in current brain-trained AI research. This absence is understandable, as using such neural signals would require sensorimotor capabilities and environmental affordances comparable, to some extent, to those accessible to the human body. In this perspective paper, we argue that humanoid robots, although not equivalent to humans, could offer a unique opportunity to leverage *embodiment-dependent neural signals*, and thereby significantly expand the range of targets available for brain-trained AI, as illustrated in Figure 1.

**Figure 1:**
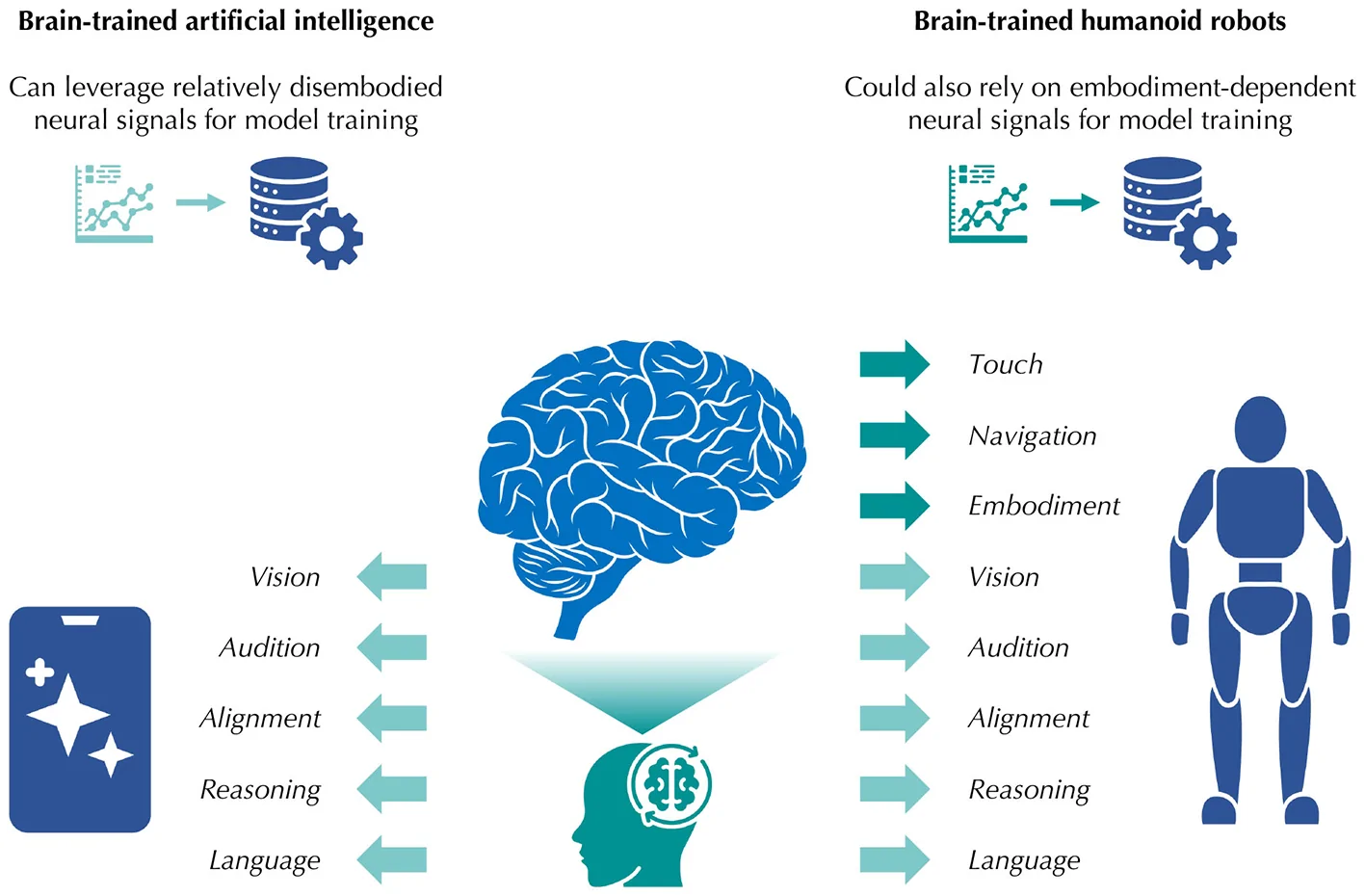
From brain-trained artificial intelligence to brain-trained humanoid robots. **Middle:** Human brain activity is the source of a diversity of neural signals, which can be recorded using various neuroimaging techniques. **Left:** Brain-trained AI can leverage relatively disembodied neural signals for model training, with experimental research demonstrating the potential of neuroimaging data in the visual, auditory, and linguistic domains, and theoretical research highlighting alignment and reasoning as two particularly promising applications for brain-trained foundation models. **Right:** Brain-trained humanoid robots could additionally rely on embodiment-dependent neural signals, such as the signals associated with touch, navigation, and other strongly embodied cognitive processes, for model training. In this sense, humanoid robots could expand the range of transferable neural representations in brain-trained AI.

## 2 Humanoid robots

The humanoid form is considered particularly desirable for robots, since it can allow robotic systems to interact with environments initially designed for humans (Sakagami et al., 2002). As humanoid robots are improved, they are expected to become increasingly capable of performing useful tasks in human environments (Kaneko et al., 2008). The humanoid form could present other advantages as well, such as a better physical interaction and non-verbal communication with humans, an improved acceptance and trust within human society, and the ability to learn from the environment in a manner that better approximates human learning (Vernon and Sandini, 2024). These capabilities build upon a series of technological advances that have gradually brought humanoid robots closer to humans in terms of dexterity, navigation, and behavior. In particular, robotic arms have become capable of performing complex (OpenAI et al., 2019) or fine (Zhao et al., 2023) manipulations, with more complete robotic systems being able to perform bimanual manipulations requiring whole-body control (Fu et al., 2024) and manipulations involving various body parts (Barreiros et al., 2025). Meanwhile, locomotion has been improved with the development of adaptable quadrupedal robots (Hwangbo et al., 2019), as well as bipedal humanoid robots capable of navigating a diversity of outdoor environments (Radosavovic et al., 2024) or learning agile sports skills (Haarnoja et al., 2024). Foundation models have opened new opportunities for robotics, with large language models (LLMs) providing semantic knowledge for robotic tasks (Ahn et al., 2022), and embodied language models (Driess et al., 2023) or vision-language-action models (Brohan et al., 2023) combining both linguistic and sensorimotor information.

Humanoid robots remain obviously very different from humans. There are, for example, anatomical differences between the morphology of the human body and the structure of humanoid robots. There are also functional differences, with human sensorimotor abilities relying on complex neural mechanisms, which are only beginning to be replicated in dedicated robotic models (Wei et al., 2024). Finally, there are developmental or evolutionary differences due to the fact that humans are the product of natural selection, with a gradual co-evolution of the human brain and sensorimotor abilities (Baker et al., 2025), whereas humanoid robots have been directly engineered to perform specific tasks. Remarkably, these differences do not appear to represent an insurmountable obstacle for leveraging embodied human supervision in robotics. Indeed, learning methods have been developed for overcoming embodiment differences between humans and robots (Liu et al., 2019), and for training agents to generalize to entirely new embodiments (Zakka et al., 2022), thereby crossing the so-called *embodiment gap* (Lum et al., 2025; Yu et al., 2025) using either visual (Lum et al., 2025) or tactile (Yu et al., 2025) demonstrations. These successful behavioral transfers suggest that the neural signals underlying these behaviors might be transferrable as well, provided that they are, like the corresponding human actions, associated with sufficiently high-level representations (Zakka et al., 2022) and not too dependent on the low-level singularities of the human body (Lum et al., 2025). As recent advances have brought humanoid robots closer to humans, we hypothesize that they have also made them increasingly compatible with the embodiment-dependent cognitive processes of the human brain, and increasingly plausible candidates for the incorporation of the corresponding neural signals. If this hypothesis is confirmed, the humanoid form could expand the possibilities of brain-trained robotics, opening a path toward brain-trained humanoid robots.

## 3 Computational strategies for brain-trained humanoid robots

Current strategies for training humanoid robots typically follow two complementary paths. The first path is to use reinforcement learning in simulated or real environments, for example by having the robots perform sensorimotor tasks (Haarnoja et al., 2024; Barreiros et al., 2025) or engage in spatial navigation (Radosavovic et al., 2024). The second path is to rely on imitation learning and guide the robots using human demonstration data, which can be acquired either by having humans teleoperate the robots (Seo et al., 2023; Murooka et al., 2025), or simply by recording humans as they freely interact with their environment (Shah et al., 2025). Brain-trained robotics represents an additional strategy, whose potential is to expand the possibilities of imitation learning by complementing human actions with neural signals. Like other applications of brain-trained AI, the objective of brain-trained robotics is to leverage neural signals that can provide useful information about human cognitive processes, beyond what is already accessible from behavioral data alone. Whereas brain-inspired AI focuses on transferring general computational principles from neuroscience to AI, with the hypothesis that these principles could improve AI systems (Zador et al., 2023), brain-trained AI focuses on transferring specific computational processes from human brains to AI models, with the hypothesis that these neural priors could help AI training. The two approaches are complementary, but they rely on distinct assumptions about neuroscience discoveries. Brain-inspired AI assumes that neuroscience has uncovered some meaningful mechanisms, which could generalize to artificial systems. By contrast, brain-trained AI assumes that the functional roles of some brain regions and processes are sufficiently well understood to extract relevant neural signals, and that these neural signals can be used to guide model training.

The first brain-trained AI assumption is not trivial: the reverse inference of a particular cognitive function from the activation of a particular brain region must be conducted with care (Poldrack, 2006), but it remains a valid operation and can have a high predictive power, especially when it is conditioned on specific tasks (Hutzler, 2014). The second brain-trained AI assumption deserves some development, as two distinct approaches have emerged to incorporate neural signals into the training strategies of AI models. In the visual and auditory domains, the alignment of models with human representations can often be relatively direct, as the training strategies can rely on the neural patterns observable in the visual cortex (Fong et al., 2018; Lu and Wang, 2025) or auditory cortex (Freteault et al., 2025) to bring the internal representations of the models closer to the observed activity of the human brain. For example, a deep convolutional neural network for object recognition can be optimized using neural patterns, by adding a multi-layer encoder that predicts fMRI signals alongside the object category; the model is then trained to minimize both the classification loss and the fMRI generation loss, effectively aligning its internal representations with the neural patterns of the visual cortex (Lu and Wang, 2025). By contrast, in the robotic domain, the alignment with human representations is arguably more indirect: rather than exploiting high-dimensional neural patterns, existing research in brain-trained robotics has essentially focused on extracting relevant low-dimensional reward and error signals, and using them to modulate the reinforcement learning process (Iturrate et al., 2010; Kim et al., 2017; Xu et al., 2021; Kar et al., 2022). The names *pattern decoding* and *cognitive and computational inference* have been proposed to distinguish these two approaches in brain-trained AI (Donoso, 2026b).

Interestingly, the opportunity to use the pattern decoding approach may depend on the functional similarity between the human brain and the AI model for the task of interest. In particular, the representational similarity observed between visual models and the visual cortex (Yamins et al., 2014), or between auditory models and the auditory cortex (Kell et al., 2018) may have facilitated a relatively direct transfer of human representations. For vision, the overlap between brain-inspired AI and brain-trained AI is particularly significant, since the visual system of mammals has directly inspired the development of convolutional neural networks (Lecun et al., 1998). Theoretically, as neuroscience research uncovers increasingly detailed functional representations in the somatosensory cortex (Wang et al., 2025) and motor cortex (Gordon et al., 2023) for sensorimotor tasks, and in the hippocampus (Crivelli-Decker et al., 2023) and entorhinal cortex (Wagner et al., 2023) for spatial navigation, experimenting with the pattern decoding approach in humanoid robotics might become increasingly plausible, especially if such neuroscience discoveries influence, in the long term, the design of the AI models dedicated to sensorimotor tasks or spatial navigation. Practically, in the short term, cognitive and computational inference may be preferred for preliminary experimentation on brain-trained humanoid robots, as it is currently the most established approach in brain-trained robotics.

### 3.1 Guiding sensorimotor tasks with uncertainty signals

One possible strategy for brain-trained humanoid robots could be to guide sensorimotor tasks using neural signals associated with uncertainty. While human demonstrators can provide examples of state-action pairs when they select a particular action *a* in a particular state *s*, the degree of confidence of the demonstrators regarding their own choices may not be easily inferred from behavioral data alone. For complex manipulations involving fragile, deformable, or irregular objects, or in other situations where sensorimotor control may be challenging, estimating the uncertainty behind each action could represent a promising solution for helping humanoid robots distinguish the more confident human choices from the more hesitant ones. For a concrete illustration, let us consider the following Equation 1:


$$\begin{array}{l} \begin{array}{cc} J\left(\theta \right)={𝔼}_{\left(s,a\right)\sim D}[(1-\sigma \left(B^{\text{uncertainty}}\left(s,a\right)\right)) & \log\pi_{\theta }\left(a|s\right)] \end{array} \end{array}$$ (1)

In this formulation, *J*(θ) represents the policy optimization objective and π_θ_ the sensorimotor policy. The policy is trained on the state-action pairs (*s, a*) of a demonstration dataset *D* in order to maximize the log-likelihood term logπ_θ_(*a*∣*s*), in line with classical imitation learning methods (Ross et al., 2011). The novelty resides in the introduction of *B*^uncertainty^, a scalar derived from an uncertainty-related neural signal associated with each state-action pair, and mapped to the [0, 1] interval through a sigmoid function. The introduction of this term modulates the policy optimization objective based on human brain activity, by down-weighting the log-likelihood term when uncertainty is high. Potentially, this strategy could allow humanoid robots to rely more cautiously on human examples when the demonstrators are not confident enough about their own choices. The functional network engaged in sensorimotor tasks could provide several promising targets for deriving *B*^uncertainty^ from human brain activity. In particular, the somatosensory cortex of mammals can encode uncertainty in sensory processing (Shin et al., 2025), while the activity of the human motor cortex varies with the degree of certainty (Tzagarakis et al., 2010) even when the actions are identical (Wiesman et al., 2021). Uncertainty-related patterns have also been uncovered in the posterior parietal cortex (PPC) (Schach et al., 2022) and cerebellum (Baumann and Mattingley, 2014), possibly opening alternative ways to align humanoid robots with human sensorimotor representations. Ultimately, the utility of neural signals should be evaluated relative to the information that can be obtained directly from behavioral data. In particular, uncertainty signals may be considered valuable if they provide an advantage over behavioral estimates of uncertainty, based for example on reaction times, movement variability, pauses, or corrective movements. This advantage could be an improved performance, but it may also be a reduced overhead when training humanoid robots for a diverse set of tasks, if neural signals emerge as a more generalizable form of supervision and lower the need for task-specific behavioral metrics. It would also be useful to compare the value of uncertainty signals with the information that can be obtained through explicit human feedback, such as confidence reports, since the difficulty to acquire some forms of human feedback has been one of the motivations advanced for brain-trained robotics (Xu et al., 2021).

### 3.2 Guiding spatial navigation with surprise signals

Another possible strategy for brain-trained humanoid robots could be to guide spatial navigation using neural signals associated with surprise. Much like the degree of confidence, the degree of surprise of the demonstrators regarding the outcome of their own choices may not be easily inferred from behavioral data alone. For complex environments involving human-populated, changing, or unstructured spaces, or in other situations where navigational control may be challenging, estimating the surprise after each movement could represent a promising solution for helping humanoid robots distinguish the more predictable locations from the more unexpected ones. For a concrete illustration, let us consider the following Equation 2:


$$\begin{array}{l} \begin{array}{cc} J\left(\theta \right)={𝔼}_{\left(s,a\right)\sim D} & [(1+\delta B^{\text{surprise}}\left(s,a\right))\log\pi_{\theta }\left(a|s\right)] \end{array} \end{array}$$ (2)

In this formulation, *J*(θ) represents again the policy optimization objective, while π_θ_ represents the navigation policy. Like earlier, the policy is trained on the state-action pairs (*s, a*) of a demonstration dataset *D* in order to maximize the log-likelihood term logπ_θ_(*a*∣*s*). This time, the novelty resides in the introduction of *B*^surprise^, a scalar derived from a surprise-related neural signal associated with each state-action pair, and whose weight is controlled by a positive parameter δ. The introduction of this term modulates again the policy optimization objective based on human brain activity, but this time by up-weighting the log-likelihood term when surprise is high. Potentially, this strategy could allow humanoid robots to learn more decisively from human examples when the demonstrators are surprised by the outcome of their own choices, as these scenarios are likely rarer and more informative than others. The functional network engaged in spatial navigation could provide several promising targets for deriving *B*^surprise^ from human brain activity, as novelty-related patterns have been uncovered in both the hippocampus (Kaplan et al., 2014) and the hippocampal-entorhinal system (Maass et al., 2014), possibly providing several paths to align humanoid robots with human navigational representations. As previously, the utility of neural signals should ultimately be evaluated relative to the information that can be obtained directly from behavioral data. In particular, surprise signals may be considered valuable if they provide an advantage over behavioral estimates of surprise, based for example on state-transition statistics or exploratory behavior. Similar to the uncertainty case, it would also be useful to compare the value of surprise signals with the information that can be obtained through explicit human feedback. In both equations, the neural signals are assumed to be appropriately preprocessed and normalized before being used.

## 4 Neural signals for brain-trained humanoid robots

Current brain-trained AI research relies on a variety of neuroimaging techniques, each with distinct strengths and weaknesses. For example, fMRI provides a high spatial resolution but requires the subject to remain immobile inside the scanner, whereas EEG offers a high temporal resolution and can be integrated into wearable devices. The possibility to combine virtual reality with fMRI (Doeller et al., 2010) or MEG (Roberts et al., 2019) can expand the possibilities of these non-portable neuroimaging techniques, and could be a promising path to explore in the context of brain-trained robotics. Nevertheless, portable neuroimaging techniques such as EEG or functional near-infrared spectroscopy (fNIRS) would probably be the preferred solutions for capturing neural signals in a diversity of real-world situations. Regardless of the specific modality, neuroimaging data, represented by B^*^(*t*) in the framework of brain-trained foundation models, should only be considered as an approximation of *B*(*t*), the true brain activity. The acquisition of usable neural signals can be a difficult problem, considering the relatively low signal-to-noise ratio, high individual variation, non-trivial correspondence between neural signals and cognitive processes, and other challenging characteristics often encountered in neuroimaging data. Importantly, these characteristics do not preclude the effective incorporation of neural signals, as demonstrated by previous successes in brain-trained robotics (Iturrate et al., 2010; Kim et al., 2017; Xu et al., 2021; Kar et al., 2022). However, they call for a targeted use of neuroimaging data for strategically chosen humanoid robot functions, where human actions *A*(*t*), such as explicit feedback, could be either difficult to acquire or too superficial as a training signal. Here, we expand on our previous computational examples and present six such functions, approximately ordered by increasing degree of abstraction: sensorimotor tasks, spatial navigation, sensorimotor integration, embodied alignment, embodied reasoning, and social integration. For each of these functions, we indicate which brain regions could realistically be targeted for neural signal extraction, and discuss how these embodiment-dependent neural signals could be leveraged to improve humanoid robots, as summarized in Table 1.

**Table 1:** Opportunities for brain-trained humanoid robots.

Humanoid robot functions	Brain regions of interest	Neural signal recording contexts	Potential neuroimaging techniques	Opportunities for humanoid robots
**Sensorimotor tasks**	Somatosensory cortex, motor cortex, PPC, cerebellum	Complex manipulations	fMRI/MEG, EEG/fNIRS	Aligning with human sensorimotor representations
**Spatial navigation**	Hippocampus, entorhinal cortex	Complex environments	fMRI/MEG with virtual reality, EEG/fNIRS	Aligning with human navigational representations
**Sensorimotor integration**	PPC, premotor cortex, SMA	Complex movements	EEG/fNIRS, possibly PET	Aligning with human integrative representations
**Embodied alignment**	Ventral striatum, vmPFC, OFC, insula	Embodiment-dependent valuation	fMRI/MEG with virtual reality, EEG/fNIRS	Aligning with embodied human values and preferences
**Embodied reasoning**	DLPFC, ACC, FPC	Embodiment-dependent execution	fMRI/MEG with virtual reality, EEG/fNIRS	Reasoning under similar or related constraints
**Social integration**	TPJ, mPFC, STS	Social interactions	fMRI/MEG, EEG/fNIRS	Aligning with human social representations

The six humanoid robot functions (sensorimotor tasks, spatial navigation, sensorimotor integration, embodied alignment, embodied reasoning, and social integration) are presented across four important dimensions (brain regions of interest, neural signal recording contexts, potential neuroimaging techniques, opportunities for humanoid robots). The table presents only a selection of the possible brain regions of interest, recording contexts, and neuroimaging techniques that could be considered.

As we discuss embodiment-dependent neural signals, it should be noted that there is currently no well-established, universal definition of embodiment. Rather, definitions can be ambiguous and overlapping (Brock, 2024), and in some areas of neuroscience, models of cognition span a continuum from completely disembodied to strongly embodied theories (Meteyard et al., 2012). For the purpose of this perspective paper, we propose to distinguish three functional levels of embodiment, approximately ordered, like the proposed humanoid robot functions themselves, by increasing degree of abstraction. The first level, which could be called *sensorimotor embodiment*, corresponds to the idea that cognitive processes and their underlying neural activity depend on sensorimotor interactions with the environment (Engel et al., 2013), and that the development of sensorimotor representations could be relevant for robotics as well (Jacquey et al., 2019). The second level, which could be called *higher-order cognitive embodiment*, extends the idea to higher-order cognitive functions (Barsalou, 2008) such as conceptual representations (Kiefer and Pulvermüller, 2012), mental imagery (Muraki et al., 2023), or memory (Buzsḱi and Tingley, 2023), while also suggesting that such embodied cognitive processes could be useful for robotics (Pezzulo et al., 2013). Finally, the third level, which could be called *social embodiment*, extends the idea from individual cognition to social cognition (Niedenthal et al., 2005; Goldman and Vignemont, 2009), with potential implications for future advances in human-robot interaction (Cross et al., 2019). The six proposed humanoid robot functions can be viewed as corresponding to relatively distinct levels in this classification, with sensorimotor tasks, spatial navigation, and sensorimotor integration building upon sensorimotor embodiment; embodied alignment and embodied reasoning relying primarily on higher-order cognitive embodiment; and social integration depending primarily on social embodiment. Naturally, this classification should only be understood as a high-level framework for organizing the discussion about relevant neural signals, as in complex real-world settings, embodied cognitive processes of different natures are often deeply intertwined.

### 4.1 Sensorimotor tasks

In the human brain, sensorimotor tasks engage a functional network including the somatosensory cortex (Ruben et al., 2001), the motor cortex (Hari et al., 1998), the PPC (Andersen and Cui, 2009), and the cerebellum (D'Mello et al., 2020). In humanoid robot research, the development of effective sensorimotor abilities stands out as a particularly difficult problem (Murooka et al., 2025), with approaches relying on tactile feedback increasingly demonstrating their potential (Barreiros et al., 2025; Zheng et al., 2026). Here, we see a clear opportunity to leverage neural signals from the corresponding brain regions, in order to improve the sensorimotor capabilities of humanoid robots—much like neural signals from the visual cortex have been used to optimize visual models (Fong et al., 2018; Lu and Wang, 2025), or neural signals from the auditory cortex to optimize auditory models (Freteault et al., 2025). Specifically, brain activity could be recorded while human subjects engage in complex manipulations, involving for example fragile, deformable, or irregular objects. The neural signals could then serve to regularize, fine-tune, or guide model training in any appropriate way, thereby aligning humanoid robots with human sensorimotor representations, as illustrated earlier in our first computational example. In this context, non-portable neuroimaging techniques such as fMRI or MEG could probably be used to some extent, alongside portable techniques such as EEG or fNIRS.

### 4.2 Spatial navigation

In the human brain, spatial navigation engages a functional network including the hippocampus (Maguire et al., 2000) and the entorhinal cortex (Doeller et al., 2010). In humanoid robot research, the implementation of robust navigation abilities remains a significant challenge (Zhang et al., 2026), particularly when robots share their environment with humans and must implement a form of human-aware navigation (Pyaraka et al., 2026). Here, as previously, we see a clear opportunity to use neural signals from the corresponding brain regions, in order to improve the navigational capabilities of humanoid robots. Specifically, brain activity could be recorded while human subjects navigate complex environments, involving for example human-populated, changing, or unstructured spaces. The neural signals could then be exploited to align humanoid robots with human navigational representations, as illustrated earlier in our second computational example. In this context, the combination of virtual reality with non-portable neuroimaging techniques could be particularly promising, alongside the use of portable techniques for real-world environments.

### 4.3 Sensorimotor integration

In the human brain, sensorimotor integration engages a functional network including the PPC, the premotor cortex (Grafton et al., 1997), and the supplementary motor area (SMA) (Nachev et al., 2008). The acquisition of a body schema, allowing robots to control their body state in a diversity of situations, is one of the current frontiers in humanoid robot research (Kawaharazuka et al., 2025). An interesting solution could be to build upon the neural signals from the corresponding brain regions, in order to improve the integrative capabilities of humanoid robots. Specifically, brain activity could be recorded while human subjects engage in complex movements, combining for example sensorimotor tasks with simultaneous spatial navigation. The neural signals could then provide a basis for aligning humanoid robots with human integrative representations. In this context, portable neuroimaging techniques would probably be preferable, or even necessary for a large range of whole-body movements, but innovative solutions may eventually expand the applications of usually non-portable techniques. Of particular interest here, a new positron emission tomography (PET) motion-compatible prototype has recently been used to record the brain activity of walking human subjects (Siva et al., 2024), suggesting that brain-trained AI might, in the future, benefit from the unique insights on neural metabolism provided by PET data in a broader range of situations.

### 4.4 Embodied alignment

In the human brain, valuation engages a functional network including the ventral striatum (Pessiglione et al., 2007), the ventromedial prefrontal cortex (vmPFC) (Lebreton et al., 2009), the orbitofrontal cortex (OFC) (Plassmann et al., 2007), and the insula (Craig, 2009). Remarkably, existing research in brain-trained robotics has essentially focused on reward and error signals, which belong arguably to the category of valuation signals. However, these signals have typically been recorded while the human brain acts as an observer of robotic behavior, for example when the subject evaluates the movements of a virtual robot (Iturrate et al., 2010). A natural possibility could be to expand the range of valuation processes considered, and include embodiment-dependent neural signals that could be used to improve the alignment of humanoid robots. Whereas sensorimotor tasks, spatial navigation, and sensorimotor integration focus on a relatively well-defined objective—such as manipulating an object, reaching a destination, or maintaining a functional body state—humans routinely engage in more open-ended behaviors, such as deciding which object to manipulate or which destination to reach. These behaviors rely on higher-order cognitive functions such as conceptual representations, mental imagery, or memory, and they are guided not only by concrete physical and spatial affordances, but by abstract valuation processes that determine which action is preferable in a particular context. This higher degree of abstraction corresponds to our earlier distinction between sensorimotor embodiment and higher-order cognitive embodiment, and it may be a desirable property for humanoid robots. Much like the text generated by an LLM is expected to align with human values, the actions of a humanoid robot in open-ended contexts should probably be optimized for human preferences as well, and neural signals could help us advance toward this objective. Specifically, brain activity could be recorded while human subjects engage in activities requiring embodiment-dependent valuation, such as selecting their actions or choosing how to modulate the execution of their movements beyond the immediate sensorimotor requirements of the task. The neural signals could then serve to align humanoid robots with embodied human values and preferences, a process which we could call *embodied alignment*. In this context, both portable and non-portable neuroimaging techniques could be considered, the latter possibly combined with virtual reality. While alignment could emerge as one of the most promising applications for brain-trained AI in general (Donoso, 2026b), embodied alignment could open new horizons for humanoid robots in particular, as it could improve their ability to follow human values and preferences in real-world situations, and help them integrate more seamlessly into human environments.

### 4.5 Embodied reasoning

In the human brain, execution engages a functional network including the dorsolateral prefrontal cortex (DLPFC) (Koechlin et al., 2003), the anterior cingulate cortex (ACC) (Behrens et al., 2007), and the frontopolar cortex (FPC) (Boorman et al., 2009). A promising direction could be to consider a range of processes associated with human executive function, and leverage embodiment-dependent neural signals to improve the reasoning capabilities of humanoid robots. While open-ended human behaviors depend on valuation processes to determine the goals to pursue, they also require executive processes to plan appropriate sequences of actions, such as successively manipulating several objects or reaching various intermediate destinations. These behaviors are guided not only by concrete physical and spatial affordances, but by abstract executive processes that determine which action could be the most effective at a particular step of a sequence. Again, this degree of abstraction may be a desirable property for humanoid robots, particularly when the pursuit of their goals is likely to require multiple steps. Much like the reasoning process of an LLM is expected to build upon a series of relevant reasoning steps, the actions of a humanoid robot in open-ended contexts should probably be optimized for step-by-step effectiveness as well, and neural signals could provide a basis for such an optimization. Specifically, brain activity could be recorded while human subjects engage in activities requiring embodiment-dependent execution, such as planning a sequence of actions under real-world physical or biological constraints. The neural signals could then be exploited to train humanoid robots to reason under similar or related constraints, a process which we could call *embodied reasoning*. In this context, both portable and non-portable neuroimaging techniques could again be considered, the latter possibly combined with virtual reality. Alongside alignment, reasoning could emerge as one of the most promising applications for brain-trained AI in general (Donoso, 2026b), although it may require the acquisition of dedicated neuroimaging datasets (Donoso, 2026c). Embodied reasoning could open new horizons for humanoid robots in particular, as it could improve their ability to execute and plan actions in real-world situations, and help them perform useful tasks in uncertain, changing, and open-ended human environments.

### 4.6 Social integration

In the human brain, social integration engages a functional network including the temporo-parietal junction (TPJ) (Carter and Huettel, 2013), the medial prefrontal cortex (mPFC) (Buckner et al., 2008), and the superior temporal sulcus (STS) (Allison et al., 2000). The development of natural human-robot interactions is one of the current frontiers in humanoid robot research (Valls Mascaro and Lee, 2025), with approaches enabling robots to perform more natural gaze shifts (Wei et al., 2026), as well as more agile and generalizable interaction skills (Wang et al., 2026). While our previous discussion on embodied alignment and embodied reasoning does not necessarily require a direct human-robot interaction, social integration adds a new degree of complexity by specifically considering the relations between several human or robotic agents. This transition from individual cognition to social cognition corresponds to our earlier distinction between higher-order cognitive embodiment and social embodiment. Social integration may present specific challenges due to the open-ended nature of human interactions, which are often highly complex and unfold over time following unpredictable trajectories (Hari et al., 2015). The fact that the neural processes underlying social cognition depend continuously on the actions, emotions, and inferred intentions of interacting agents has motivated the emergence of second-person neuroscience (Redcay and Schilbach, 2019) and hyperscanning methods (Czeszumski et al., 2020). Nevertheless, the interaction scenarios relevant for humanoid robots are currently more limited than the full range of possible human interactions, which suggests that carefully chosen neural signals could still be useful to improve the social capabilities of humanoid robots. Specifically, brain activity could be recorded while human subjects engage in well-defined forms of social behavior, requiring for example directed attention, emotion recognition, or theory of mind. The objective would not be to investigate the full range of possible human interactions, but to align humanoid robots with a meaningful set of human social representations. In this context, non-portable neuroimaging techniques could probably be used to some extent, alongside portable techniques. It is worth noting that valuation, execution, and social capabilities could probably complement each other in many real-world situations, suggesting the possibility to incorporate simultaneously a variety of neural signals into the training strategies of humanoid robots.

## 5 Neural signal extraction

The two computational examples presented earlier illustrate that neural signals might be incorporated into the training strategies of humanoid robots by extending classical imitation learning methods, without necessarily requiring the development of entirely new architectures. This suggests that in the foreseeable future, the primary challenge for brain-trained humanoid robots would probably reside more in the extraction of relevant neural signals, at an appropriate level of precision, in order to meaningfully complement established training pipelines. Existing research in brain-trained robotics has successfully leveraged reward and error signals acquired using EEG, which is a relatively noisy neuroimaging technique, suggesting that some classes of signals, at least, can be effectively extracted despite a low signal-to-noise ratio. It seems reasonable to assume that the level of precision required could depend on the strength or robustness of the neural signals, with signals associated with weaker or more subtle effects potentially requiring a higher spatial or temporal resolution, or a higher signal-to-noise ratio. The accuracy and reliability of the extraction could depend on the spatial or temporal structure as well, with some distributed or complex signals possibly requiring dedicated neuroimaging methods. This could motivate preliminary experimentation on brain-trained humanoid robots to focus on relatively robust and well-established neural signals, such as uncertainty or surprise signals, before considering more challenging possibilities. Experimenting with the pattern decoding approach could also require a higher level of precision, as high-dimensional neural patterns may necessitate a more fine-grained recording than low-dimensional neural signals.

Although we primarily focused on non-invasive neuroimaging modalities such as fMRI, MEG, EEG, and fNIRS, invasive techniques such as intracortical arrays or intracranial EEG (iEEG) could also prove useful, as these techniques can allow for the acquisition of neural signals at a substantially higher resolution and with an excellent signal-to-noise ratio. Of particular interest here, intracortical arrays implanted in the motor cortex have been used to control a robotic (Hochberg et al., 2012) or virtual (Serino et al., 2022) arm, and arrays implanted in the somatosensory cortex to provide tactile stimuli (Flesher et al., 2021), while iEEG has been used to investigate neural signals associated with spatial navigation (Seeber et al., 2025; Lv et al., 2025) or abstract representations (Courellis et al., 2024) in the hippocampus. Invasive techniques have already been leveraged in brain-trained AI to improve a deep learning model for a decision-making task (Aquino et al., 2025), suggesting that they might provide valuable information for brain-trained humanoid robots as well. Different variants of iEEG may serve different purposes, with electrocorticography (ECoG) potentially being a good compromise for extracting cortical signals, and stereoelectroencephalography (sEEG) remaining particularly useful for subcortical regions. At this level of precision, a successful transfer may depend, as we discussed earlier, on whether the neural signals are associated with sufficiently high-level representations, and not too dependent on the low-level singularities of the human body.

Importantly, invasive recordings are currently less practical than non-invasive neuroimaging modalities, as they are usually restricted to clinical settings, rendering them less scalable as a potential source of information for brain-trained humanoid robots. We suggest that invasive recordings might still emerge as a useful complement to non-invasive neuroimaging modalities in some specific contexts, such as optimizing humanoid robots for particularly common and relevant sensorimotor or navigational behaviors. Indeed, as the number of human demonstrators is expected to be low, neural signals acquired through invasive techniques would probably be more relevant for improving a selected set of widely shared behaviors, while non-invasive neuroimaging modalities could remain the preferred method for spanning a more diverse range of behavioral conditions, where generalization from a few examples would be more difficult to achieve. The development of implantable brain-computer interfaces could gradually expand the contexts in which invasive recordings are acquired, potentially increasing their utility for brain-trained humanoid robots.

## 6 Challenges and opportunities

The most fundamental technical challenge for brain-trained humanoid robots could be the fact that the humanoid form, although intended as an approximation of the human body, remains significantly different in terms of sensorimotor capabilities. This could render the transfer of neural representations from the human brain to humanoid robots non-trivial, and possibly more difficult than similar attempts in the visual, auditory, or linguistic domains, further motivating the consideration of relatively robust and well-established signals, such as uncertainty or surprise, for preliminary experimentation. As the technology of humanoid robots continues to advance, it would be particularly useful to investigate the correspondence between models trained for sensorimotor tasks and the corresponding regions of the human brain, such as the somatosensory and motor cortices, especially if new architectures influenced by neuroscience discoveries are eventually developed. This would allow us to explore whether the representational similarity observed between visual models and the visual cortex (Yamins et al., 2014), or between auditory models and the auditory cortex (Kell et al., 2018), holds for sensorimotor models despite the singularities of the humanoid form, possibly providing some insights regarding the feasibility of the pattern decoding approach. Another challenge could be the relative scarcity of the neuroimaging datasets acquired to investigate touch, compared to those dedicated to vision or audition. Leveraging pre-trained models to generate neuroimaging data at scale could be an interesting solution, as this approach has already been used in brain-trained AI (Zhao et al., 2025), but it would still require that these models are trained on existing tactile datasets. On the other hand, it is worth noting that research on brain-trained humanoid robots could potentially be valuable beyond the full humanoid form, as some neural representations may be transferrable to robotic arms, robotic heads, or other partial imitations of the human body.

Another opportunity could be to use brain-trained humanoid robots as embodied computational models of human cognition, thereby expanding the potential of brain-trained foundation models for cognitive simulation (Donoso, 2026a) by including embodiment-dependent neural signals. Existing research has already highlighted the possibility to use brain-trained visual (Hansen et al., 2025) or auditory (Moussa et al., 2025) models to gain insights into the cognitive processes of the human brain, suggesting that brain-trained humanoid robots could serve this objective as well. This possibility could be particularly valuable for neuroscience research, as the computational processes underlying the sensorimotor system are arguably less understood than the computational processes of the visual or auditory systems. In the short term, the cognitive and computational inference approach could give immediate indications about the kind of neural signals that can effectively guide humanoid robots for sensorimotor tasks, spatial navigation, or other relevant functions, possibly providing some insights regarding the robustness and plausibility of the underlying neuroscience models. In this sense, brain-trained humanoid robots could serve as an experimental platform for evaluating scientific hypotheses about strongly embodied cognitive processes. In the long term, if the pattern decoding approach proves successful for brain-trained humanoid robots, bringing the internal representations of their models closer to the neural representations of the human brain, an even more forward-looking opportunity might emerge: using brain-trained humanoid robots as embodied generators of synthetic neuroimaging data, by predicting neural patterns based on their internal representations. Naturally, these predicted neural patterns would only be imperfect approximations of actual human brain activity, and they might not necessarily generalize to novel situations outside their training data distribution. Nevertheless, the possibility to generate synthetic neuroimaging data at scale in real-world situations could still be valuable for neuroscience research on the sensorimotor system, much like foundation models for vision, audition, or language are currently developed to accelerate scientific discovery in the corresponding domains (d'Ascoli et al., 2026).

Regarding the ethical and social challenges, the most distinctive risk for brain-trained humanoid robots could be excessive anthropomorphism. If successful, the incorporation of embodiment-dependent neural signals into the training strategies of humanoid robots may contribute to increase their perceived humanity, possibly creating a mismatch between the expectations of human subjects and the actual cognitive and emotional capabilities of humanoid robots. This problem could be complex to address, in particular because of the complex nature of the anthropomorphism phenomenon itself. Indeed, this phenomenon simultaneously depends on physical embodiment, social behavior, and communication skills, and it can either facilitate acceptance among humans, or on the contrary, evoke rejection when the anthropomorphic form is overused without being supported by human-like capabilities (Fink, 2012). This raises the question of the degree of anthropomorphization that should be deliberately pursued when developing brain-trained humanoid robots. As a general principle, we propose that the incorporation of neural signals could be useful when it improves the functions of humanoid robots without harming the necessary transparency about their robotic nature. Existing research on anthropomorphization highlights the value of transparency as a design principle, and suggests that transparent robotic designs can improve the effectiveness of robots for particular tasks (Zhong et al., 2023). While humans acquire their knowledge through their experience of embodied interactions with the world, humanoid robots trained on human brain data should not wrongly suggest that they have developed their own capabilities in the same manner. The development of machines capable of learning and thinking like humans is a fundamental research question (Lake et al., 2017), but this objective remains quite far from the reality of current foundation models, which acquire their representations by being trained on a large corpus of existing data. Although ensuring an appropriate degree of anthropomorphization may represent a challenge, it should be considered in light of the significant opportunities that could be offered by brain-trained humanoid robots, such as a better alignment with human expectations and values, and the ability to perform useful tasks in uncertain, changing, and open-ended human environments.

## 7 A new strategy for humanoid robots

In the *Robot* series, science fiction writer Isaac Asimov invented the concept of a “positronic” brain for humanoid robots. Intriguingly, research on brain-trained humanoid robots may take this fictional device one step closer to reality, as neural signals extracted from the human brain could narrow the distance between humanoid robotics and human neuroscience. Regardless of the specific neuroimaging modality considered, this new strategy for humanoid robots could significantly expand the range of transferable neural representations in brain-trained AI, by leveraging embodiment-dependent neural signals that cannot be meaningfully exploited in disembodied AI systems. These neural signals could potentially improve the capabilities of humanoid robots in the context of sensorimotor tasks, spatial navigation, sensorimotor integration, embodied alignment, embodied reasoning, and social integration, opening new challenges and opportunities for human-robot interaction. Humanoid robots are not designed to be equivalent to humans, and the risk of excessive anthropomorphism that may arise from this strategy should be addressed responsibly. Nevertheless, as we gradually expand the range of human cognitive experiences that could be directly used to train models, we may reinforce the alignment of AI systems with the aspirations and endeavors of humankind. Eventually, we might even discover whether humanoid robots themselves dream of electric—or positronic—brains.

## Data Availability

The original contributions presented in the study are included in the article/supplementary material, further inquiries can be directed to the corresponding author.
